# Supplementary material for: A Potential ABA Analog to Increase Drought Tolerance in Arabidopsis thaliana
Source: Int J Mol Sci. 2023 May 15;24(10):8783. doi: 10.3390/ijms24108783 (PMC10218568; doi:10.3390/ijms24108783)
Supplement: Supplementary file 1 [file ijms-24-08783-s001.zip › ijms-2356361-supplementary.pdf]

A Potential ABA Analog to Increase Drought Tolerance in *Arabidopsis thaliana*

Ruiqi Liu [1,2,†] and Guoyan Liang [1,2,†], Jiaxin Gong [1,2], Jiali Wang [1,2], Yanjie Zhang [1,2], Zhiqiang Hao [1,2], Guanglin Li [1,2,*]

[1] Key Laboratory of Ministry of Education for Medicinal Plant Resource and Natural Pharmaceutical Chemistry, College of Life Sciences, Shaanxi Normal University, Xi'an 710119, China

[2] National Engineering Laboratory for Resource Development of Endangered Crude Drugs in Northwest China, College of Life Sciences, Shaanxi Normal University, Xi'an 710119, China

* Correspondence: glli@snnu.edu.cn

† These authors contributed equally to this work.

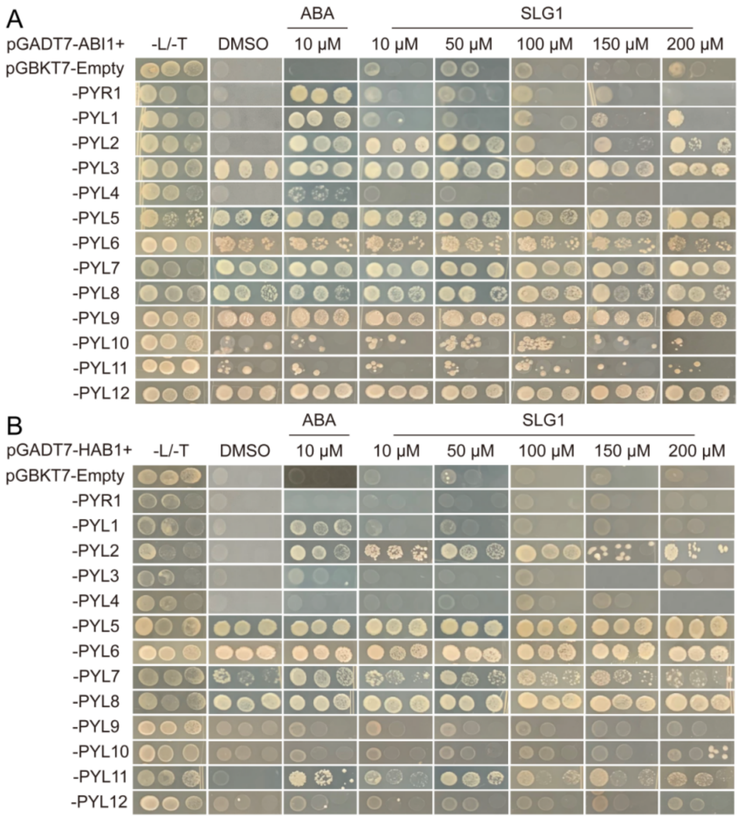

Figure S1. SLG1 is a potent agonist of multiple ABA receptors for ABI1 and HAB1. Yeast-two-hybrid assay showing the interactions of the binding domain (BD)-fused PYR1/PYLs with the activation domain (AD)-fused ABI1 (A) or HAB1 (B) on SD/−Leu/−Trp/−His (−L/−T/−H) media containing (+)-ABA and corresponding compounds. (+)-ABA was used at 10 μM concentration and SLG1 at 10 to 200 μM concentrations. SD/−Leu/−Trp (−L/−T) were used to check whether the plasmid has been transferred into the yeast strain AH109. SD/−Leu/−Trp/−His media containing 0.1% DMSO was used as negative control. Three replicates were maintained per concentration.

Table S1. Results of 10 pharmacophores.

Pharmacophores	Feature	Rank	Direct Hit	Partial Hit	Max Fit
01	HHAA*	26.136	111	000	4
02	HHAA	25.503	111	000	4
03	HHAA	24.568	111	000	4
04	HHAA	24.5611	111	000	4
05	HHAA	24.460	111	000	4
06	HHAA	24.314	111	000	4
07	HHAA	24.284	111	000	4
08	HHAA	24.264	111	000	4
09	HHAA	24.176	111	000	4
10	HHAA	24.120	111	000	4

*H represents the hydrophobic center, and A represents the hydrogen bond acceptor.

Table S2. Parameters of 10 small molecular compounds identified via virtual screening.

Name	Code	Structure	Molecular Weight	LibDock Score	FitValue	-CDOCKER Interaction energy
SLG1	JFD00519	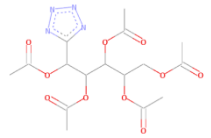	430.37	121.476	1.9868	67.0954
SLG2	JFD01598	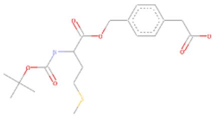	397.49	124.628	3.61764	57.9197
SLG3	BTB13322	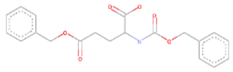	371.39	129.188	2.72517	60.6008
SLG4	BTB15187	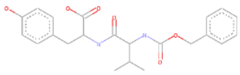	414.46	133.845	2.69074	55.0674
SLG5	JFD01601	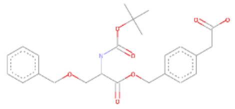	624.82	123.148	3.12546	55.0674
SLG6	NRB01725	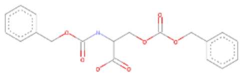	373.36	124.019	2.6105	68.2586
SLG7	PD00593	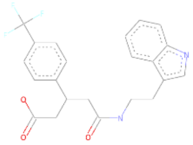	418.42	131.544	3.26145	56.501
SLG8	RJC00839	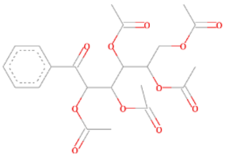	466.44	120.4211	3.2158	55.4447
SLG9	RJC02528	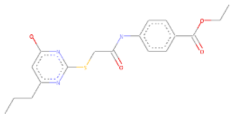	375.44	120.188	3.2231	55.543
SLG10	RJC00899	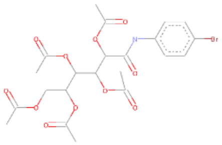	560.35	123.151	2.72971	63.0988

Table S3. Primers used in the study.

Gene	Primer sequence (5'-3')	Purpose
Actin2	F: AGCACTTGCACCAAGCAGCATG<br>R: ACGATTCCTGGACCTGCCTCATC	
P5CS1	F: CGATAGCTTAGCTGCTCTACTGGCGT<br>R: AGCTGCATTGACTGCAGCTTTGACT	
RD29b	F: TGTCTTCTGACCACACCAAACCCATTG<br>R: CCACCAGGAGCAAACGTCCTAGTCA	
ABF2	F: TTGACTCTGCCTCGAACGCTTAG<br>R: CCTCTGACTCTGACTCTGACTCTG	qRT-PCR
KIN1	F: TGGAGCTGGAGCACAACA<br>R: GACCCGAATCGCTACTTGTTC	
ABI1	F: ACGTACCAGATTACGCTCATATGATGGAGGAAGTATCTCCGGCG<br>R: CAGCTCGAGCTCGATGGATCCGTTCAAGGGTTTGCTCTTGAGTT	
ABI2	F: ACGTACCAGATTACGCTCATATGATGGACGAAGTTTCTCCTGCAGT<br>R: CAGCTCGAGCTCGATGGATCCGGTTCTCAATTCAAGGATTTGCTCTTGAATTTCC	
HAB1	F: GCCATGGAGGCCAGTGAATTCATGGAGGAGATGACTCCCGCA<br>R: CAGCTCGAGCTCGATGGATCCGGTTCTGGTCTTGAACTTTCTTTG	
PYL1	F: TCAGAGGAGGACCTGCATATGATGGCGAATTCAGAGTCCTCCTC<br>R: TCGACGGATCCCCGGGAATTACCTAACCTGAGAAGAGTTGTTG	pGADT7
PYL2	F: TCAGAGGAGGACCTGCATATGATGAGCTCATCCCCGGCCGT<br>R: TCGACGGATCCCCGGGAATTATTCATCATCATGCATAGGTGC	
PYL3	F: TCAGAGGAGGACCTGCATATGATGAATCTTGCTCCAATCCATGATC<br>R: TCGACGGATCCCCGGGAATCAGGTCGGAGAAGCCGTG	
PYL4	F: TCAGAGGAGGACCTGCATATGATGCTTGCCGTTCACCGTCCTTC<br>R: TCGACGGATCCCCGGGAATCACAGAGACATCTTCTTCTTGC	
PYL5	F: TCAGAGGAGGACCTGCATATGATGAGGTCACCGGTGCAACTCC<br>R: TCGACGGATCCCCGGGAATTATTGCCGGTTGGTACTTCGAG	
PYL6	F: TCAGAGGAGGACCTGCATATGATGCCAACGTCGATACAGTTTC<br>R: TCGACGGATCCCCGGGAATTACGAGAATTTAGAAGTGTTCTCGG	
PYL7	F: TCAGAGGAGGACCTGCATATGATGGAGATGATCGGAGGAGAC<br>R: TCGACGGATCCCCGGGAATCAAAGGTTGGTTTCTGTATGATTC	
PYL8	F: TCAGAGGAGGACCTGCATATGATGGAAGCTAACGGGATTGAG<br>R: TCGACGGATCCCCGGGAATTAGACTCTCGATTCTGTCGTG	
PYL9	F: TCAGAGGAGGACCTGCATATGATGATGGACGGCGTTGAAGGC<br>R: TCGACGGATCCCCGGGAATCACTGAGTAATGTCCTGAGAAG	pGBKT7
PYL10	F: TCAGAGGAGGACCTGCATATGATGAACGGTGACGAAACAAAG<br>R: TCGACGGATCCCCGGGAATCATATCTTCTTCTCCATAGATTC	
PYL11	F: TCAGAGGAGGACCTGCATATGATGGAAACTTCTCAAAAATATCATAC<br>R: TCGACGGATCCCCGGGAATTACAACTTTAGATGAGCCACCC	
PYL12	F: TCAGAGGAGGACCTGCATATGATGAAAACATCTCAAGAACAGCATG<br>R: TCGACGGATCCCCGGGAATTAAGTGAGCTCCATCATCTTCTCCG	
HAB1	F: GATCTGGTTCCGCGTGGATCCATGGAGGAGATGACTCCCGCAG<br>R: GATGCGGCCGCTCGAGTCGACTCAAAAGAAGCAATGCTTACCGATGG	
ABI1	F: GATCTGGTTCCGCGTGGATCCATGGAGGAAGTATCTCCGGCG<br>R: GATGCGGCCGCTCGAGTCGACTCAGTTCAAGGGTTTGCTCTTGAG	
ABI2	F: GATCTGGTTCCGCGTGGATCCATGGACGAAGTTTCTCCTGCAG<br>R: GATGCGGCCGCTCGAGTCGACTCAATTCAAGGATTTGCTCTTGAATTTCC	
PYR1	F: CAGCAAATGGGTCGCGGATCCATGCCTTCGGAGTTAACACCAGAAG<br>R: TGCGGCCGCAAGCTTGTCGACTCACGTCACCTGAGAACCACTTC	pGEX-4T-1
PYL1	F: CAGCAAATGGGTCGCGGATCCATGGCGAATTCAGAGTCCTCCTC<br>R: TGCGGCCGCAAGCTTGTCGACTTACCTAACCTGAGAAGAGTTGTTG	
PYL2	F: CAGCAAATGGGTCGCGGATCCATGAGCTCATCCCCGGCCGT<br>R: TGCGGCCGCAAGCTTGTCGACTTATTCATCATCATGCATAGGTGC	
PYL3	F: CAGCAAATGGGTCGCGGATCCATGAATCTTGCTCCAATCCATGATC<br>R: TGCGGCCGCAAGCTTGTCGACTCAGGTCGGAGAAGCCGTG	
PYL4	F: CAGCAAATGGGTCGCGGATCCATGCTTGCCGTTCACCGTCCTTC<br>R: TGCGGCCGCAAGCTTGTCGACTCACAGAGACATCTTCTTCTTGC	
PYL5	F: CAGCAAATGGGTCGCGGATCCATGAGGTCACCGGTGCAACTCC<br>R: TGCGGCCGCAAGCTTGTCGACTTATTGCCGGTTGGTACTTCGAG	
PYL6	F: CAGCAAATGGGTCGCGGATCCATGCCAACGTCGATACAGTTTC<br>R: TGCGGCCGCAAGCTTGTCGACTTACGAGAATTTAGAAGTGTTCTCGG	pET28a
PYL8	F: CAGCAAATGGGTCGCGGATCCATGGAAGCTAACGGGATTGAG<br>R: TGCGGCCGCAAGCTTGTCGACTTAGACTCTCGATTCTGTCGTG	
PYL7	F: GGCGGCCGCGATATCGTCGATGGAGATGATCGGAGGAGAC<br>R: TTACCTGCAGGGAATTCGGTCAAAGGTTGGTTTCTGTATGATTC	
PYL9	F: GGCGGCCGCGATATCGTCGATGATGGACGGCGTTGAAGGC<br>R: TTACCTGCAGGGAATTCGGTCACTGAGTAATGTCCTGAGAAG	
PYL10	F: GGCGGCCGCGATATCGTCGATGAACGGTGACGAAACAAAG<br>R: TTACCTGCAGGGAATTCGGTCATATCTTCTTCTCCATAGATTC	
PYL11	F: GGCGGCCGCGATATCGTCGATGGAAACTTCTCAAAAATATCATACG<br>R: TTACCTGCAGGGAATTCGGTTACAACTTTAGATGAGCCACCC	pMal-c5x
